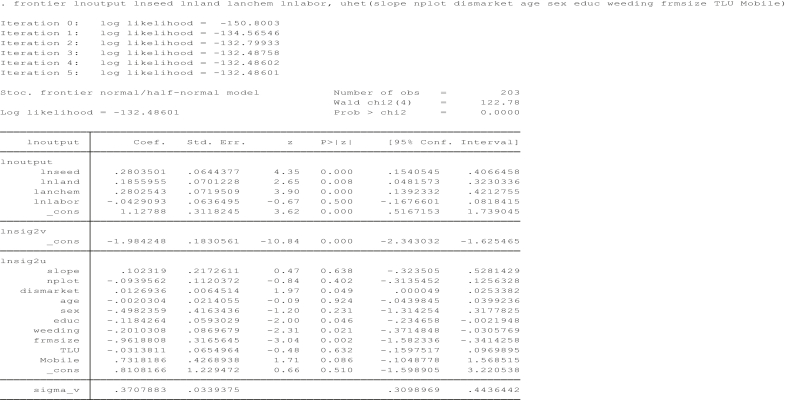# Are farmers technically efficient in growing sorghum crops?: evidence from western part of Ethiopia Gudeya Bila district

**DOI:** 10.1016/j.heliyon.2022.e09907

**Published:** 2022-07-08

**Authors:** Tolesa Tesema

**Affiliations:** Lecturer at Wollega University Shambu Campus, Ethiopia

**Keywords:** Smallholder farmers, Sorghum production, Stochastic frontier, Technical efficiency

## Abstract

Agriculture provides the majority of Ethiopian rural households with their principal source of income, yet it performs poorly. There is a rise in food insecurity as well as a decline in productivity as a result of this. Even if sorghum production in Ethiopia is increasing rapidly, it requires an efficient level of output to ensure high levels of productivity and profit. Hence the goal of this study was to examine the technical efficiency of sorghum production and its determinants in the Gudeya Bila area in western Ethiopia, utilizing primary data obtained through semi-structured questionnaires from 203 randomly selected households. The study utilized one-stage stochastic frontier production model to investigate the technical efficiency and its determinants. The mean technical efficiency of the homes was 45.64 percent, according to the results of the stochastic frontier of the parametric approach. These results suggest that farmers in the research area are technically inefficient in sorghum by 56.36 percent on average. Weeding frequency, farm size, and cell phone use were also key factors of technical efficiency in a one-stage stochastic frontier approach. As a result, the study reveals that by enhancing technological efficiency, it may be possible to increase production to the level of potential output. Ensure mobile information service, raise knowledge about intensive land use, subsidize chemical inputs, and expand educational possibilities in the research region are some of the numerous strategies to improve technical efficiency.

## 1 Introduction

Developing countries including Ethiopia transforming rural development can sustainably diminish poverty (Diao et al., 2012). Despite this, agricultural productivity is failing due to a steadily increasing population with and systems of production (Getachew, 2020). More ever the sector is characterized by a very large number of fragmented landholdings and vagaries relying mainly upon the summer rains in the presence of a lack of cultivatable land available (United State Aid, 2020). The government intervention and financial and labor market development affect resource reallocation heterogeneously (Zheng and Ma, 2022). Hence resource use inefficiency agriculture are the major causes of deforestation, environmental pollution, and land degradation worldwide (Joneydi, 2012). Ethiopia were the country with ingenious home of sorghum and is the source of many wild and cultivated forms adapted to a wide range of growing conditions, especially in drought areas, valued more for home consumption purposes such as human food, as fuel, as building materials, and as feed for livestock (CSA, 2019; Kate and Leigh, 2010). Thus, sorghum consumption is driven by consumers replacing sorghum for teff in injera as teff was estimated at 5.3 million metric tons in 2019/20, up by 200,000 metric tons (United State departments of agriculture, 2020). more ever comparatively, the average productivity of sorghum in Ethiopia is 2.1 tons/hectare, which is far below the global average of 3.2 tons/hectare due to the problems of drought, striga, insect pests, diseases, soil fertility decline, inadequate adoption of existing improved varieties, lack of high yielding and good quality sorghum varieties (Kinfe and Tesfaye, 2018). Due to its inherent nature, sorghum has drought-resistant mechanisms that make it a better fit in moisture-stressed areas and less competitive with other crops (Agricultural transformation agency, 2020). Towards this end, the productivity of sorghum was also hindered by the use of local drought-tolerant but low-yielding landraces because farmers had been forced to abandon high-yielding and late-maturing landrace cultivars because of the frequent occurrence of drought (Assefa et al., 2016). Indeed, sorghum is a major cereal crop in the research area, second only to maize, but its productivity is low. Incognizant to this farmers' knowledge and performance, as well as production limits, must be included from the beginning stages of breeding and technological development to improve sorghum yield (CSA, 2019). More ever one approach for satisfying current and future demand on a sustainable basis without endangering future land resource usage is to increase productivity and more optimal and efficient resource use (Friew, 2015). Toward this the factors influencing the farm performance were investigated including technical efficiency by mechanization levels (Huan et al., 2022; Minah et al., 2022; Zhou and Ma 2022) improved agricultural technologies and practices (Mohammed and Abdulai 2022; Nonvide 2021; Owusu et al., 2020; Setsoafia et al., 2022; Zheng et al., 2021a), and information technologies (Huang and Khan 2022; Ma and Zheng 2022; McFadden et al., 2022). However, little is known about how the introduction of drought-tolerant crops affects technical efficiency. As a result, this work addresses a vacuum in the literature by conducting a sorghum-specific analysis. This helps to emphasize the importance of this research. Furthermore, small farms dominate crop agriculture in Ethiopia's many regions and agro-ecologies for both their own use and sales (Alemayehu et al., 2012; Duguma and Han 2020). The sorghum farming system used in the current study in Gudeya Bila district was different from that used in other areas that used oxen. The following procedure is used to accomplish this. First, forest must be cleared from farmland using hand-hoe farm implements without the use of oxen. The forest was drayed on the property next to the clearing, and the fire was set in the drayed forest. They began to cultivate sorghum on that field after the forest burned on the land and soon after the rain rained on the land. As a result of the above-mentioned unique farming technique of drought-tolerant sorghum crop in the Gudeya Bila district, western Ethiopia, there was a research vacuum in the current work. Furthermore, this research will improve farmers' livelihood activities by recognizing policy choices, increasing resource use efficiency, and providing more intensive development support in such farming systems in the study area in particular and for policymakers in general. The goal of this study was to evaluate the levels of technical efficiency in sorghum production and to investigate the factors influencing those levels in the Gudeya Bila district of western Ethiopia.

## 2 Reviews of the literature

### 2.1 Theoretical literature

Precision agriculture efficiency increases are likely to be cumulative in terms of technical efficiency (Delay et al., 2022). A producer is deemed technically efficient if they achieve the highest potential output from their inputs (Coelli et al., 1998). One of the most crucial aspects of the manufacturing process is efficiency. Technical efficiency in the manufacturing process refers to a company's capacity to manufacture goods with the least amount of waste. When technical and allocative efficiency are combined, the result is economic efficiency, also known as overall efficiency (Coelli et al., 1998). Using technical efficiency, we may compare the observed and optimal amounts of output and inputs of a production unit (Coelli, 1995). It's calculated by comparing the actual output to the possible (border) output (Fried et al., 2008; Tutulmaz, 2014). Lovell (1993) defines a production unit's efficiency as the difference between observed and ideal output and input values. The ratio of observed to maximum potential output obtainable from the given input, or the ratio of minimum potential to observed input required to create the given output, can be used to make the comparison. The optimum is defined in terms of production possibilities in these two comparisons, while efficiency is technical. According to Kumbhakar and Lovell (2003), technical efficiency is attained when the firm can produce a maximum level of outputs given a certain level of inputs or minimize inputs given a certain level of outputs. Battese and Coelli (1988) employed the production frontier function and a one-stage estimate approach for inefficiency effects models in their investigation. The stochastic frontier is the most appropriate method for efficiency studies that account for inefficiency issues as well as technical faults that arise during both measurement and observation (Coelli et al., 1998). Promoting Internet use in rural areas can help remedy these errors and improve farm productivity (Zheng et al., 2021b).

### 2.2 The study's conceptual framework

Food consumption is currently expanding at a faster rate than before due to population growth.

**Figure 1:**
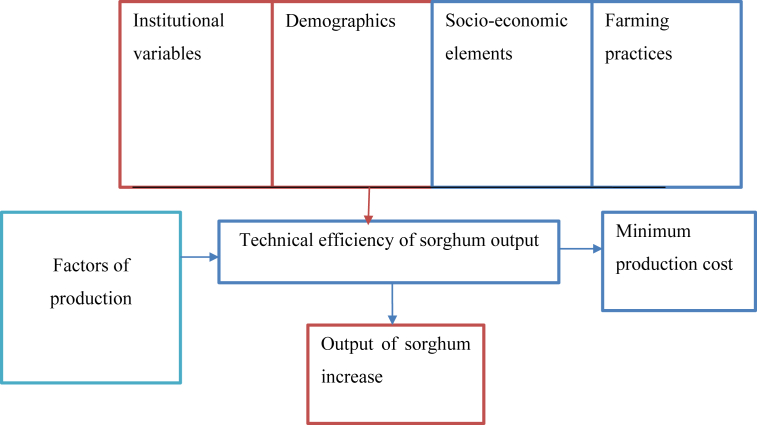
Conceptual framework of the study. Source: The author's design.

Thus motivating the importance of farmers for sustainable agricultural production and development meet the growing demand for food (Dagar et al., 2021) (Dagar et al., 2021). A production frontier indicates the maximum output that can be produced under different input amalgamations; the ratio of the unit's output to the maximum possible output gives a measure of efficiency (Ephraim, 2014; Bicknell and Renwick 2019) (Ephraim, 2014; Bicknell and Renwick 2019). The two main goals of the stochastic frontier are to estimate the underlying production technology and to measure household-specific technical inefficiency (Kumbhakar and Sun 2013). Additionally Sustainable financial development and innovations increases efficiency (Zakari et al., 2022). As a result, the efficiency with which inputs are translated into outputs is determined by the inputs used, as well as a variety of socioeconomic and institutional aspects, as well as farm features (Jema, 2006; Battese and Coelli, 1988). As a result, improving the socio-economic, farm, institutional, and resource ownership characteristics of farmers is a prerequisite for increasing production efficiency (James, 2010). The conceptual framework is also used in Figure 1 below, which depicts how various factors interact to influence smallholder farmers' drought-tolerant sorghum crop efficiency in the study area. As a conceptual framework for this research study, this scenario is represented graphically.

## 3 Material and methods

### 3.1 Description of study area

This research was conducted in the Gudeya Bila districts in western Ethiopia. Land structures in the Gudeya Bila district are undulating. Dystric Nitosol, which has considerable agricultural potential, dominates the district. Land clearing is done in March, April, and May, which is the start of the rainy season, according to the district's agricultural calendar. The average annual minimum and maximum temperatures are 11.300 °C and 23.360 °C, respectively. The wet season runs from May to September, whereas the dry season is from October to April. The annual rainfall averaged between 1400 and 2000 mm. It has an area of 842.75 square kilometers (84275 ha) (Gudeya Bila Woreda, Office of Agriculture and Natural Resources, 2021). Farmers in the lowlands generate income by growing sorghum, which is characterized by a rain-fed production system, no oxen plowing, and no automated farming.

### 3.2 Sampling techniques and data used

Multi stage sampling techniques were used for present study. In the frist stage the Gudeya Bila were selected purposively from Ethiopia because the researchers were convenience to the study area and the sorghum were the dominant crop in study area for livelihoods. In second stage the three kebeles of the district, namely Abay Dale, Gute Chancho, and Zangi, were selected from Gudeya bila district due to the reason that sorghum were the dominant crop in those kebeles. Lastly after researchers know the sorghum producers, data was collected from 203 sample households. Sample size was determined by Eq. (1) below the formula provided.

(1) $$n=\frac{N}{1+\left(e^{2}\right)N}$$

where n is sample size which were 203 and N is the numbers of maize producers household in the district which is 44890 at 7% levels of precision (e).

Before gathering data, the researchers obtained informed consent from Gudeya Bila office of agriculture and natural resource and consult with development agents to learn about the sorghum farming system. After that the semi-structured questionnaires prepared by the researchers were distributed to sorghum producer sample households. The questionnaires were adjusted throughout data collection on qualitative and quantitative information, which is why semi-structured was employed in this study. Because oxen power was not considered an input for sorghum production in the study area, the variables oxen power used in questionnaires created at the researcher's office were eliminated. Rather than plowing with oxen, the farmer plants seeds in the earth, which germinate. Then, except for oxen power and socioeconomic and farm characteristics, the data acquired by questionaries' questions covers sorghum farm output and the inputs utilized in the production process, such as land, labor, seed, and chemicals.

### 3.3 Analytical model used

The Cobb-Douglas functional form, according to Coelli (1995), has the most appealing aspect, which is its simplicity. Furthermore, the Translog production function is more difficult to estimate and has significant estimating issues. One of the estimation issues is that as the number of variable inputs grows, so does the number of parameters to be estimated (Huan et al., 2022).

Another issue is that the additional terms require input variables to be cross-produced, which leads to considerable multicollinearity and degrees of freedom concerns. Even though the Cobb-Douglas model assumes unitary substitution elasticity, constant production elasticity, and constant factor demand, if the goal is to analyze efficiency measurement rather than the general structure of the production function, it provides adequate representation of technology and has little impact on efficiency measurement (Coelli et al., 2005). Another issue is that the additional terms require input variables to be cross-produced, which leads to considerable multicollinearity and degrees of freedom concerns. Even though the Cobb-Douglas model assumes unitary substitution elasticity, constant production elasticity, and constant factor demand, if the goal is to analyze efficiency measurement rather than the general structure of the production function, it provides adequate representation of technology and has little impact on efficiency measurement (Coelli et al., 2005). Additionally previous researches show that both stochastic frontiers model In other words, only panel data are better suited to handle the statistical noise and measurement error when stochastic frontier model and data envelopment analysis models are applied respectively (Ruggiero, 2007). In the absence of panel data, we therefore choose SFM in this study. Hence following Aigner et al. (1977) and Meeuse and van Den Broeck (1977), the cobb Douglas stochastic frontier model is defined as Eq. (2) used for current study:

(2) $$\begin{array}{l} \ln\;Y_{i}=\beta_{0}+\beta_{1}\;\ln\;x_{1i}+\beta_{2}\;\ln\;x_{2i}+\beta_{3}\;\ln\;x_{3i}+\beta_{4}\ln_{4i}+e_{i}\\ e_{i}=V_{i}-U_{i} \end{array}$$

where

ln-denotes the natural logarithm

*j*-represents the number of inputs used to produce drought-tolerant sorghum in the study area.

*i*-represents the i^th^ drought-tolerant sorghum producers in the study area.

*Y*_*i*_-is observed drought-tolerant sorghum output of the i^th^ farmer in production season.

X_1i_ = amount of local seed used in kilogram for drought-tolerant sorghum.

X_2i_ = area of land allocated for drought-tolerant sorghum production in hectare.

X_3i_ = chemical such as herbicides and pesticides used for drought-tolerant sorghum production in a liter.

X_4i_ = labor used for drought-tolerant sorghum production in man-day

*β*_0*,*_*β*_4-_ stands for parameters to be estimated for inputs

*e*_*i*_-is a composed disturbance term made up of two elements (*V*_*i*_ - *U*_*i*_). The random error *V*_*i*_ -accounts for the stochastic effects beyond the farmer's control such as (weather, pest, and diseases), and measurement errors and *U*_*i*_ captures the technical inefficiency effects. Following Battese and Coelli (1988) the specification of the technical inefficiency model as Eq. (3) as follows.

(3) $$u_{i}=\delta_{0}+\sum_{1}^{10}\delta_{i}x_{i}+v_{i}$$

*u*_*i*_-is the technical inefficiency of the i^th^ drought-tolerant sorghum producers and is assumed to be a function of farm-specific socio-economic and farm management practices.

*δ*_0_-Intercept term of technical inefficiency model.

*δ*_1_,...*δ*_10_-are the coefficient of parameter estimates of the technical inefficiency variables.

*x*_*i*_- Stand for vectors of farmers specific variables that affect the technical inefficiency of the ith farmers of drought-tolerant sorghum, such as slope dummy (steep or flat), number of plots measured in number, distance to the market in a minute, household ages in a year, sex of household dummy (male or female), education in the year farmers learn in school, weeding frequency in number, farms size in a hectare, livestock holding in a tropical livestock unit, and (1 if used and 0 if not). wi was a randomly distributed random variable with a normal distribution.

Farmers growing drought-tolerant sorghum crops confront inefficiency as well as a survey technical error. For this work, the stochastic frontier model was used to account for the implications of these errors. As a result, a single-step estimating approach was employed in this study to quantify the extent of technical inefficiency while also identifying factors affecting technical inefficiency in sorghum production. Based reviews of past scholar (Table 1) the model is specified as Eq. (4) as follows.

(4) $$\begin{array}{l} \ln output\;ofsorghum=\beta_{ο}+\beta_{1}\;\ln\;seed+\beta_{2}\;\ln\;land+\\ \beta_{3}\;\ln\;chemicals+\beta_{4}\;\ln\;labor+vi++\delta_{1}slope+\\ \delta_{2}number\;ofplot+\delta_{3}dis\;\tan\;ce\;to\;the\;market+\delta_{4\;}age+\;\\ \delta_{5}sex+\delta_{6}education+\delta_{7}weeding\;frequency+\\ \delta_{8}farm\;size+\delta_{9}livestock\;holding+\delta_{10}mobile+u_{i} \end{array}$$

**Table 1:** Reviews on determinants of technical efficiency.

Variables	Sign	Authors
Slope of land (dummy)	+	Nguyen et al. (2022)
Number of plot (dummy)	+	Jirarud and Suwanmaneepong (2020)
Distance to market in minute	-	Dessie et al. (2020)
Age of household in year	+	Skevas and Grashuis (2020)
Sex of household (dummy)	+	Okoror and Areal (2020)
Education levels in year of schooling	+	Dagar et al. (2020)
Weeding frequency in number	+	Anang et al. (2022)
Farm size in hectare	+	Dourandish et al. (2020)
Livestock holding in Tropical livestock	+	Lemma et al. (2020)
Uses of mobile (dummy)	+	Khan et al. (2019)

## 4 Result and discussion

### 4.1 Half normal model estimation of maximum likelihood estimation

The maximum-likelihood estimates of the inputs used in the stochastic production frontier model and inefficiency effect models were analyzed by the stochastic frontier model in a one-stage process. Out of the total four input variables considered in the production function, only three inputs (seed, land, and chemicals) had a significant effect in explaining the variation in sorghum production among farmers. Based on these findings, the coefficient of input is interpreted as elasticity. The results revealed that sorghum farmers had positive decreasing returns to scale (return to scale = 0.703) in sorghum production, which indicated that sorghum production was in the rational stage of production (Stage II). This shows farmers had the possibility of increasing inputs used to attain maximum output. The nature of returns to scale obtained in this study compares favorably with a similar study by Oladeebo and Ambe (2007). The gamma value of 0.756 suggested that 75.92% variation in output was due to the differences in technical efficiencies of farm household in study area while the remaining 24.08% was due to the effect of the disturbance term.

**Amount of seed applied per kilogram:** Seed is the proxy for germination of crops and is an important variable that explains the sorghum output in the study area. Despite this, farmers in the study area use local seeds that produce low output when compared with improved sorghum varieties. Benson et al. (2014) show that problems relating to the timeliness of seed delivery and the quantity and quality of seed provided are common in Ethiopia. As per information obtained from the key informant interview, the major focus of the dissemination of seed varieties is more on other crops such as maize, wheat, and teff in the study area than sorghum. This shows there is less concern about the dissemination of the improved seed variety in the study area. Coefficients of seed were significant at a 1% level of significance, which shows that as seed-applied per kilogram increases by 1%, the yield of sorghum output increases by 28% up to the optimum utilization of seed. Because the seed is above the optimum application rate, the bulk of seedlings at germination stages results in a decline in sorghum production (Table 2).

**Table 2:** Results of maximum likelihood estimation one stage.

Input variables	Coefficient	Standard error	Z	P > z
logarithm of seed	0.280∗∗∗	0.064	4.35	0.000
logarithm of land	0.185∗∗∗	0.070	2.65	0.008
logarithm of herbicide and pesticides	0.280∗∗∗	0.071	3.90	0.000
logarithm of Labor	-0.042	0.063	-0.67	0.500
Constant	1.127∗∗∗	0.311	3.62	0.000
Inefficiency variables				
Slope of land	0.102	0.217	0.47	0.638
Number of plot	-0.093	0.112	-0.84	0.402
Distance to market	0.012∗∗	0.006	1.97	0.049
Age of household	-0.002	0.021	-0.09	0.924
Sex of household	-0.498	0.416	-1.20	0.231
Education levels	-0.118∗∗	0.059	-2.00	0.046
Weeding frequency	-0.201∗∗	0.086	-2.31	0.021
Farm allocated for crops	-0.961∗∗∗	0.316	-3.04	0.002
Livestock holding	-0.031	0.065	-0.48	0.632
Uses of mobile	0.731∗	0.426	1.71	0.086
Constant	0.810	1.229	0.66	0.510
Sigma square	0.265∗∗∗	0.0509		
Lambda	1.744			
Gama	0.759	0.293		

Source: Stochastic frontier model output: one-stage estimation approach.

**Land used for sorghum production in hectare:** In the study area, farmers produce the sorghum either on their own land or by sharing it with other households. The study done by Jin et al., 2015 showed that optimal land use management can play an important role in promoting a virtuous ecosystem cycle and agricultural productivity. The coefficient of land was significant at a 1% level of significance. This means that, as the land for sorghum increases by 1%, the output of sorghum will increase by 18.55%. This is due to the fact that every production cannot be done without land, which is the original factor of production in the study area (Table 2).

**Amount of herbicide and pesticide applies in litter**: Chemicals are the proxy variables for controlling weeds and pests in the study area. Thierry et al. (2016) found that herbicides had a positive impact on grain sorghum yields regardless of local environmental conditions. In the production of sorghum, a 1% increase in the application of chemicals can increase the level of sorghum output by 0.28 percent (Table 2).

### 4.2 Determinants of technical efficiency of sorghum

**Weeding frequency:** Weeding frequency is a proxy variable for the reduction of crop failures due to weeds and insects. As per information obtained from key informant interviews, even if some farmers use chemicals to control weeds, the different grasses cannot be destroyed by those chemicals. So it needs repeated weeding by the hand of the farmers. The coefficient of weeding frequency is significant at 5% levels of significance and negatively affects technical inefficiency. This is due to the fact that the productivity of sorghum is improved by repeatedly weeding, which in turn increases the efficiency of farmers. The result shows that as weeding frequency increases by one percent, the technical efficiency increases by 0.2%. This finding is supported by Kusse et al. (2019).

**Distance to the market:** The coefficient of distance to the market is positively and statistically significant at 5% levels of significance to technical inefficiency. This is due to the fact that, as long as farmers' houses are far from the market, the possibility of farmers getting available inputs and market information is limited. This could be attributed to the fact that the farther the market was from the respondent's residence, the greater the cost of transport and opportunity cost would be. This in turn may hinder the optimal application of farm inputs and lead to technical inefficiency. The result shows that as farmers' distance to the market increases by one unit, technical efficiency decreases by 0.012%. This study is supported by Liu et al. (2021).

**Uses of mobile phones:** The coefficients of mobile phones on technical inefficiency were negative and statistically significant at 10%. As the mobile telephone is instrumental for searching agricultural and market information results indicated that mobile telephone has created a significant impact in improving the technical efficiency of farmers. Farm households that own mobile telephones have up to be more technically efficient than those farm households that do not have. This implies that farmers that own mobile telephones are more productive than those who do not have. These results agree with findings of Renwick et al. (2018) conclude that the average technical efficiency consistently higher for mobile uses relative to their counterparts, highlighting the positive role of mobile in promoting efficient usage of production inputs and information.

**Farm allocated for multiple crops:** In the study area, farmers are engaging in the production of multiple crops rather than the specialization of only a single crop. The main advantages of large farm size are the reduced risk of crop failure and the benefits of economies of scale. So the decisions of farmers in one crop help the outputs of others. If one crop fails, another can survive. This variable is significant at a 1% level of significance, and its coefficient is negative on technical inefficiency, indicating that there is a positive relationship between farm size and the amount of plot allocated for sorghum production. The coefficient of the amount of farm size used for crop production indicates that a 1% increase in the amount of land used for multiple crop production leads to a 0.97% increase in the farmer's efficiency. This finding agree with findings of Feng et al. (2021).

**Household levels of education:** Education improves technical skills, knowledge, and adaptability to production techniques. The results of this analysis show that education level negatively and significantly affects inefficiency at 5% levels of significance. This is due to educated farmers' having the capacity to allocate both natural and manmade resources to sustain the environmental risk and produce at efficient levels. This indicates that education, rooted in human capital, enhances the productivity of households since they will be better able to allocate homemade and purchased inputs, select the appropriate quantities of purchased inputs, and chooses among available techniques. This finding agrees with the findings of (Jema and Bosena, 2018; Mekonen and Gebrezgiabher, 2021).

### 4.3 Histogram and kernel density estimate stochastic frontier model

**Figure 2:**
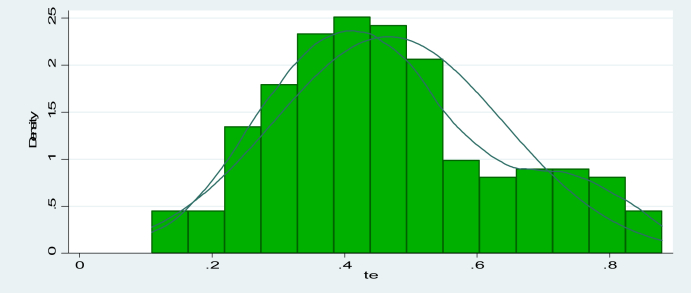
Histogram and kernel density estimate of technical efficiency in the study area. Source: computed from technical efficiency score.

The mean technical efficiency of the one-stage stochastic frontier technique was 46.64 percent, which was lower than the estimate of Mohamed and Asia (2016). To verify that the half-normal distributional assumption is met, a kernel density function is shown in Figure 2 as follows. It proves that the inefficiency impact error term is distributed in a non-negative half-normal manner.

## 5 Conclusion and policy implication

Agriculture's low productivity in Ethiopia stymies economic progress and leads to food insecurity. This is also a common occurrence in the study area that was found. As a result of this discovery, there is a significant production gap in the research area between actual and frontier output. As a result, it is necessary to improve smallholder farmers' efficiency levels by increasing the key determinants of efficiency in the study area through government agricultural policies. The key determinant inputs of production, according to the stochastic frontier model, were seed, land, and fertilizer. Because the farmers in the study area are growing sorghum with indigenous seed, additional efforts by the government and other non-governmental organizations are needed to expand capacity for intensive methods of land cultivation and work on the diffusion of improved seed types. Furthermore, because the farmers in the research region are poor, they are unable to afford the high expenses of chemical inputs imported from other countries.

Farmers must pay a lower price for chemical fertilizers as a result.

Furthermore, smallholder households growing sorghum were inefficient in terms of technology, and so there is a chance to increase efficiency by addressing several major policy factors that influenced households' technical inefficiency in the research area.

Furthermore, agricultural stakeholders must create programs to entice more young people to work in agriculture. Furthermore, roads should be built by the government to facilitate quick and inexpensive transportation from farm to market. Furthermore, given the huge potential of mobile phones in enhancing technical efficiency and production, the Ethiopian government should continue to increase information services that must be marketed to farm households via mobile phone, according to the report. The study found that weeding frequency has a positive impact on technical efficiency. Traditional weeding implements are still used by farmers in the research region. Farmers should be provided with advanced agricultural devices to control weeds on the farm in order to greatly increase their efficiency. Finally, because education is one of the most important predictors of sorghum technical efficiency, offering education opportunities in the research area through adult learning and farmer training centers was vital. More research on technical efficiency in investments farms is required done in the study area and comparative efficiency analysis across different farming system has to be done.

## Declarations

### Author contribution statement

Tolesa Tesema: Conceived and designed the experiments; Performed the experiments; Analyzed and interpreted the data; Contributed reagents, materials, analysis tools or data; Wrote the paper.

### Funding statement

This research did not receive any specific grant from funding agencies in the public, commercial, or not-for-profit sectors.

### Data availability statement

Data included in article/supp. material/referenced in article.

### Declaration of interests statement

The authors declare no conflict of interest.

### Additional information

No additional information is available for this paper.

## Figures and Tables

**Figure:**